# Aflatoxins are natural scavengers of reactive oxygen species

**DOI:** 10.1038/s41598-021-95325-8

**Published:** 2021-08-06

**Authors:** E. Finotti, A. Parroni, M. Zaccaria, M. Domin, B. Momeni, C. Fanelli, M. Reverberi

**Affiliations:** 1 Council for Agricultural Research and Economics-Food and Nutrition Center, Via Ardeatina 543, Rome, Italy; 2 Department of Environmental Biology, “Sapienza” University of Rome, P.le Aldo Moro 5, Rome, Italy; 3 Department of Biology, Boston College, 140 Commonwealth Avenue, Chestnut Hill, MA USA

**Keywords:** Chemical biology, Microbiology

## Abstract

The role of aflatoxins (AFs) in the biology of producing strains, *Aspergillus* sect. Flavi, is still a matter of debate. Over recent years, research has pointed to how environmental factors altering the redox balance in the fungal cell can switch on the synthesis of AF. Notably, it has been known for decades that oxidants promote AF synthesis. More recent evidence has indicated that AF synthesis is controlled at the transcriptional level: reactive species that accumulate in fungal cells in the stationary growth phase modulate the expression of aflR, the main regulator of AF synthesis—through the oxidative stress related transcription factor AP-1. Thus, AFs are largely synthesized and secreted when (i) the fungus has exploited most nutritional resources; (ii) the hyphal density is high; and (iii) reactive species are abundant in the environment. In this study, we show that AFs efficiently scavenge peroxides and extend the lifespan of *E. coli* grown under oxidative stress conditions. We hypothesize a novel role for AF as an antioxidant and suggest its biological purpose is to extend the lifespan of AFs-producing strains of *Aspergillus* sect. Flavi under highly oxidizing conditions such as when substrate resources are depleted, or within a host.

## Introduction

Numerous studies have investigated mycotoxins and strategies for their control, because mycotoxins’ carcinogenic and toxic effects on human and animals represent a global concern^1^. Recent outbreaks of *Aspergillus flavus* infection on maize in Europe and sub-Saharan areas^2^ have raised concern in the international community. To inform and drive strategies for mycotoxin control, researchers are invested in discovering the factors that determine mycotoxin synthesis and secretion as well as the natural role of mycotoxins in the environment.

The synthesis of secondary metabolites is thought to allow fungi to better compete against other organisms from overlapping trophic niches^3,4^. In relation to this, secondary metabolites may be toxins (AFs, ochratoxins, patulin^5^) or aggressive factors against plants (deoxynivalenol, nivalenol, fumonisin B1^6,7^) or humans (gliotoxin^8^). As for AFs, mainly produced by *Aspergillus* sect. Flavi, the ecological role is still debated. Indeed, several factors driving AF synthesis have already been successfully assessed, and oxidants and oxidative stress have been established to modulate AF synthesis. In the past decade, it has emerged that AF may represent a “metabolic response” to oxidative stress consequent to ageing or environmental insults^4,9^. Specifically, *A. flavus* may be able to tolerate reactive oxygen species (ROS) to maintain its growth, metabolism, and differentiation^10^. A recent study^11^ highlighted how “secondary ROS” produced during the enzymatic steps of AF synthesis may regulate the fitness of *A. parasiticus*. However, it is still an open debate whether AF can benefit the producer itself^4^, since among natural populations almost 50% of *A. flavus* and 10% of *A. parasiticus* do not produce AF at all^12^. Why would certain *Aspergilli* synthesize such a complex decaketide rightly regarded as a “luxury molecule”^13^?

Starting from the observation that oxygen and its reactive species represent an input for AF synthesis, we tested the hypothesis that AF acts as an antioxidant and could favor the survival of *A. parasiticus* in highly oxidizing environments. To the best of our knowledge, the intrinsic antioxidant features of AFs are here reported for the first time.

## Results

### Production of aflatoxins by *A. parasiticus* is different between open and closed systems

**Figure 1:**
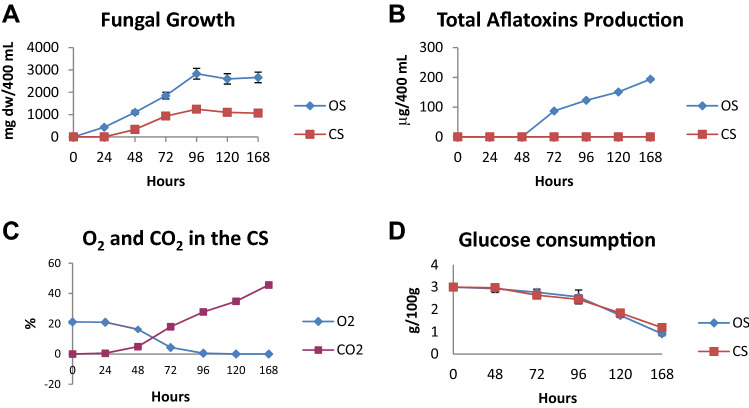
**(A)** Growth of *Aspergillus parasiticus* strain 2999 inoculated in PDB medium in the open system (OS) and closed system (CS) and incubated at 28 °C for 168 h. **(B)** Total aflatoxin production (B_1_ + B_2_ + G_1_ + G_2_) by *Aspergillus parasiticus* inoculated in PDB medium in OS and CS and incubated at 28 °C for 168 h. **(C)** Trend of the percentage (%) of O_2_ and CO_2_ during the time course of the growth of *Aspergillus parasiticus* inoculated in PDB medium in B CS and incubated at 28 °C for 168 h, in the OS the percentage of O_2_ and CO_2_ remain the same. **(D)** Analysis of glucose consumption during the growth of *Aspergillus parasiticus* inoculated in PDB medium in OS and CS and incubated at 28 °C for 168 h. Each value is the mean of three determinations ± S.D.

In an open system (OS) (Fig. 7), *A. parasiticus* presented a growth log phase peak (about 2800 mg dry weight) at 96 h after incubation (hrs) and entered the stationary phase soon after this time. In the closed system (CS) (Fig. 7), *A. parasiticus* showed growth similar to the OS, with the notable exception that it reached the late log/stationary phase 24 h earlier than the OS, i.e. at 72 h (Fig. 1A). Nevertheless, fungal growth was lower in the CS, compared to the OS (Fig. 1B). In contrast to CS in which AF was not synthesized, AF biosynthesis in the OS started at 48 h and increased up to 168 h. *A. parasiticus,* in the OS and in the culture medium employed synthesized only AF congeners B_1_ and G_1_; notably, the amount of AFG_1_ was significantly higher than that of AFB_1_ (87.1, 102, 123, 132 µg/400 mL).

This difference is possibly related to oxygen consumption and carbon dioxide accumulation (Fig. 1C. In the CS, O_2_ concentration decreased from 20 to 5% at 72 h, leading to hypoxic conditions for *A. parasiticus* as early as 96 h. As expected, the trend of CO_2_ concentration constantly increased up to 45% at 168 h of fungal growth, in the CS. In the OS, O_2_ and CO_2_ starting parameters (O_2_ = 20.58% and CO_2_ = 0.085%) stayed constant throughout growth (data not shown).

Glucose uptake registered a very similar trend in both systems (Fig. 1D), which implies a comparable metabolic energy expenditure.

### Aflatoxins demonstrate antioxidant capacity against abiotic oxidants

According to current literature and previous results, oxygen is deemed to control AF synthesis^14,15^. In an OS, *A. parasiticus* accumulates reactive species (RS) during growth, and especially during the late log/stationary phase^16^. RS are potentially toxic to the fungus itself. To counteract their effect, *Aspergillus* secretes several antioxidant compounds^17^. In our experimental setting*,* we found that a substantial amount of AFs (up to hundreds of ppb) was secreted during the late log/stationary phase. For this reason, we hypothesized that possibly AFs are countering oxidants, allowing and/or extending fungal survival during the stationary phase.

**Figure 2:**
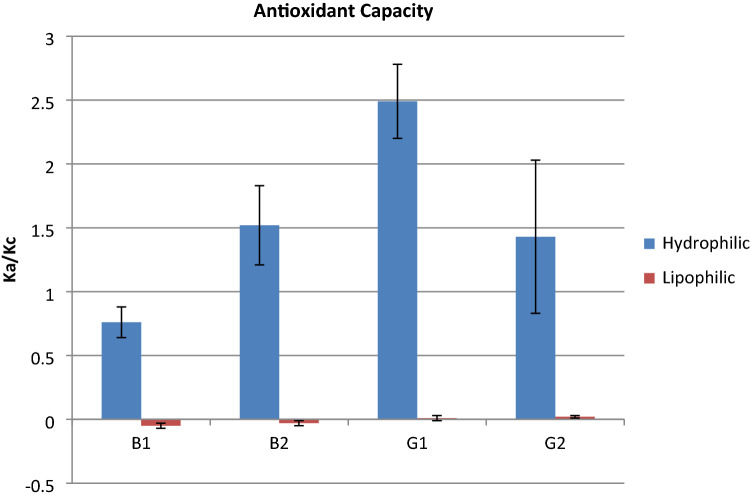
Level of aflatoxins antioxidant capacity. The result showed that, among the four aflatoxins, the antioxidant capacity was as follows: G_1_ > B_2_ > G_2_ > B_1_. Moreover, aflatoxins demonstrated a higher antioxidant capacity in the hydrophilic environment, compared to the lipophilic one. Each value is the mean of three determinations ± S.D.

To explore this possibility, we assayed the potential of the four AFs (B_1_, B_2_, G_1_ and G_2_) to scavenge RS produced by the oxidant 2,2'-Azobis, 2-amidinopropane (APAB) (see methods for details). We carried this out in hydrophilic and lipophilic environments via crocin bleaching test (Fig. 2). The result showed that, among the four AFs, the antioxidant capacity was as follows: **G**_**1**_ > B_2_ > G_2_ > **B**_**1**_ (AF produced by our strain in bold) in the hydrophilic environment. There was no or minimal antioxidant effect in the lipophilic environment. In addition, AFG_1_ presented an antioxidant value (K_a_/K_c_ = 2.49) comparable with that of the hydrophilic fraction of some polyphenols known for their important antioxidant activity^18–20^.

**Figure 3:**
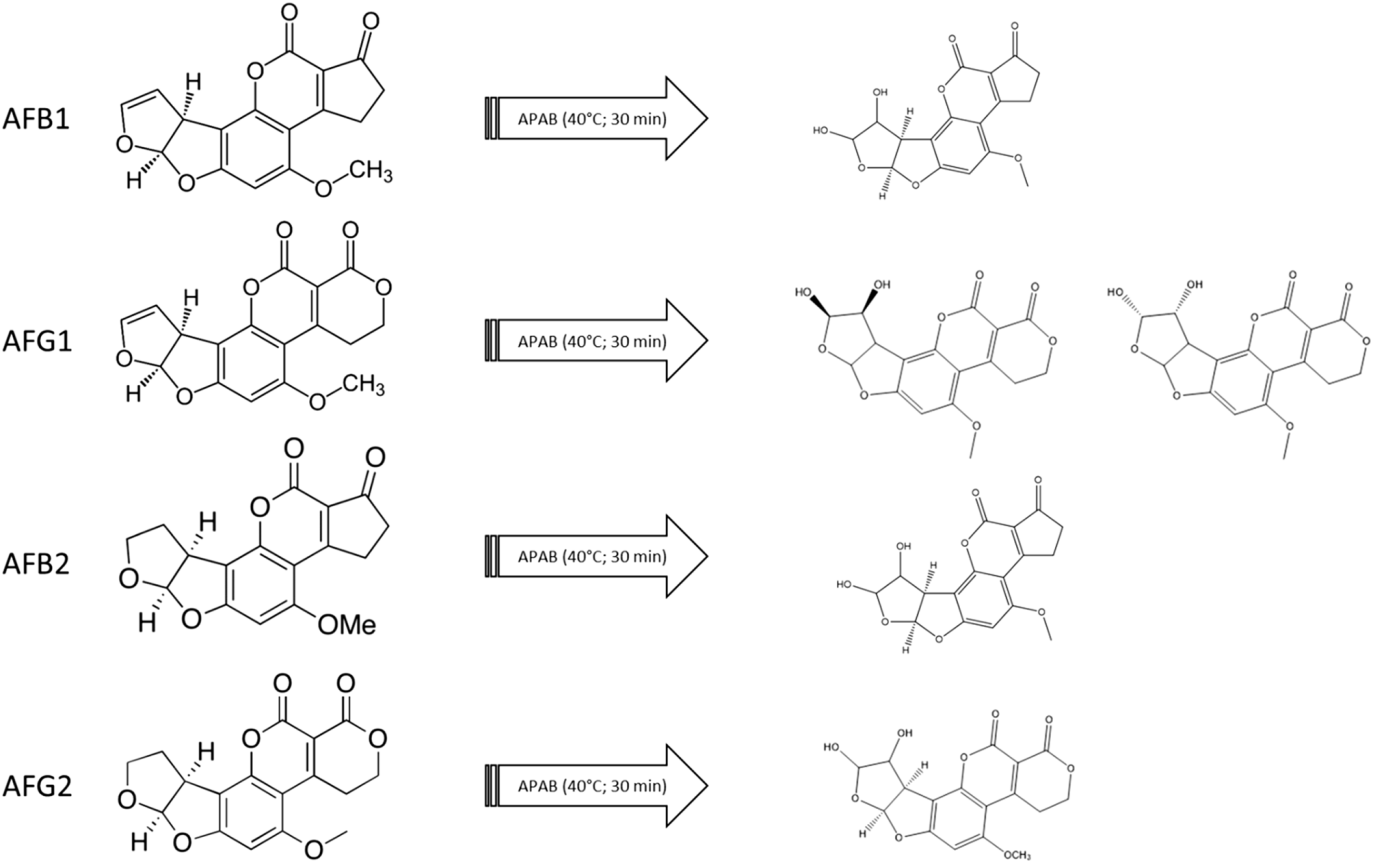
Oxidation of aflatoxins (B_1_, B_2_, G_1_, and G_2_) by APAB in a reaction performed at 40 °C for 30 min. AF byproduct’s structures were hypothesized using by accurate mass measurement.

To act effectively as an antioxidant, in presence of an oxidant AF should transform to a stable antioxidant compound^21^. Therefore, we analyzed the reaction products for the four AFs exposed to APAB oxidation. In every reaction, we recovered the dihydrodiol byproduct of AF (Fig. 3).

**Figure 4:**
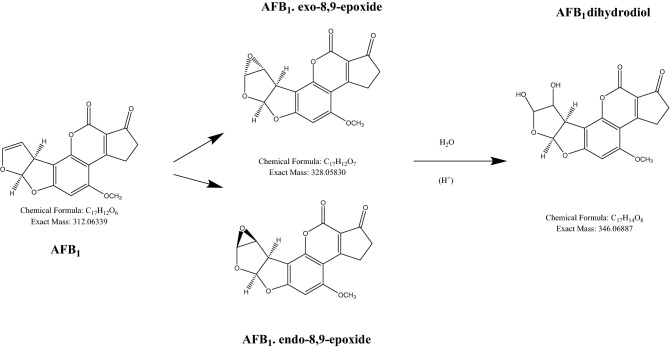
A postulated reaction scheme of AFB_1_ degradation after exposure to APAB.

In Fig. 4, we postulated the reaction scheme for AFB_1_, the most toxic of AFs. Based on known AF oxidation products, the peaks identified from the mass spectra seemed to be in agreement with the postulated reaction scheme outlined above^22^.

Although we did not see the epoxide peak in this data set, it seems reasonable for the dihydrodiol peak formation to go via epoxide. There were good mass accuracies for the AFB_1_ dihydrodiol peaks, [M + H]^+^ *m/z* 347.0766 with − 0.26 ppm error, [M + Na]^+^ *m/z* 369.058 with − 0.56 ppm error, with good mass spectral isotope matches. There was a number of peaks showing the mass *m/z* of 347, but this could be due to the formation of the dihydrodiol from the endo, exo-8,9-epoxides, resulting in the hydroxide in the different configurations; one peak may be attributed to the formation of the AFB_1_ dialdehyde.

### Aflatoxins exhibit antioxidant capacity for cultures of a model organism *Escherichia coli* K-12

**Figure 5:**
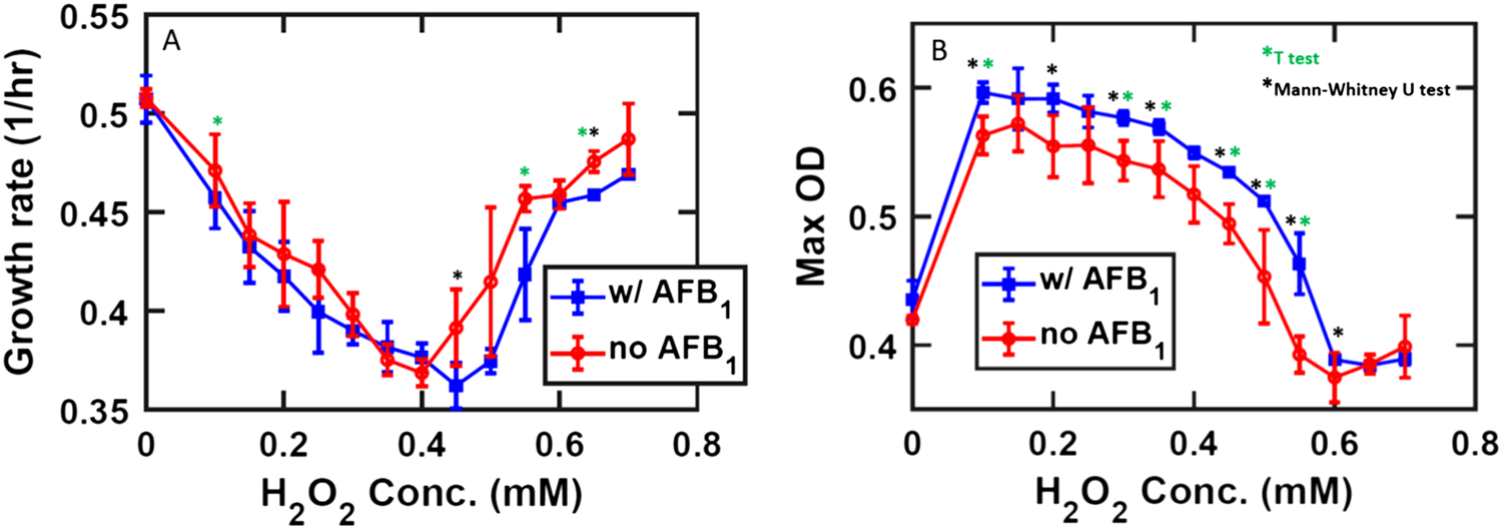
The red line represents control samples, the blue line the toxin-supplemented samples. Mean values are marked as circles, error bars are standard deviations calculated for the five experimental replicates under consideration. **(A)** Average of the growth rates at different H_2_O_2_ intervals. **(B)** Average of maximum OD. The T test and the unparametrized Mann–Whitney test (MatLab Ranksum function) have been performed, the ‘*’ represent statistically significant data points.

*E. coli* K12 was exposed to hydrogen peroxide in a 0 to 0.8 mM range. Growth rate was severely affected up to 0.45 mM, and less so beyond that threshold (Fig. 5A).

Indeed, OD values, monitored to estimate cell density, increase up to 0.1 mM H_2_O_2_ and decrease at higher concentrations (Fig. 5B). Intriguingly, the analysis showed an increase in maximum OD (Fig. 5B) for all the cultures exposed to H_2_O_2_ in the presence of 20 µg/mL AFB_1_, compared to the control. This overall advantage in population size is not a consequence of increased growth rate which, conversely, registers lower values than the control. Of notice is the reduced variability across the sample readouts of the toxin-supplemented instances: the toxin exerts a stabilizing effect on the overall cellular growth rate in presence of the oxidative stress, whereas values of growth rate and OD show more variability when the toxin is not present, as highlighted by the error bars in the graph (Fig. 5A,B). The intermediate values of H_2_O_2_ concentrations are the ones that register the highest improvement in cell viability, which progressively evens out as the H_2_O_2_ concentration approaches both the extreme intervals. In summary, the overall effect of AFB_1_ is generally beneficial to *E. coli* population abundance at the tested concentration of H_2_O_2_-elicited oxidative stress. A second experiment was performed to compare AFB_1_ effect on *E. coli* K12 cultures in comparison to the antioxidant Phenol red (PhR).

**Figure 6:**
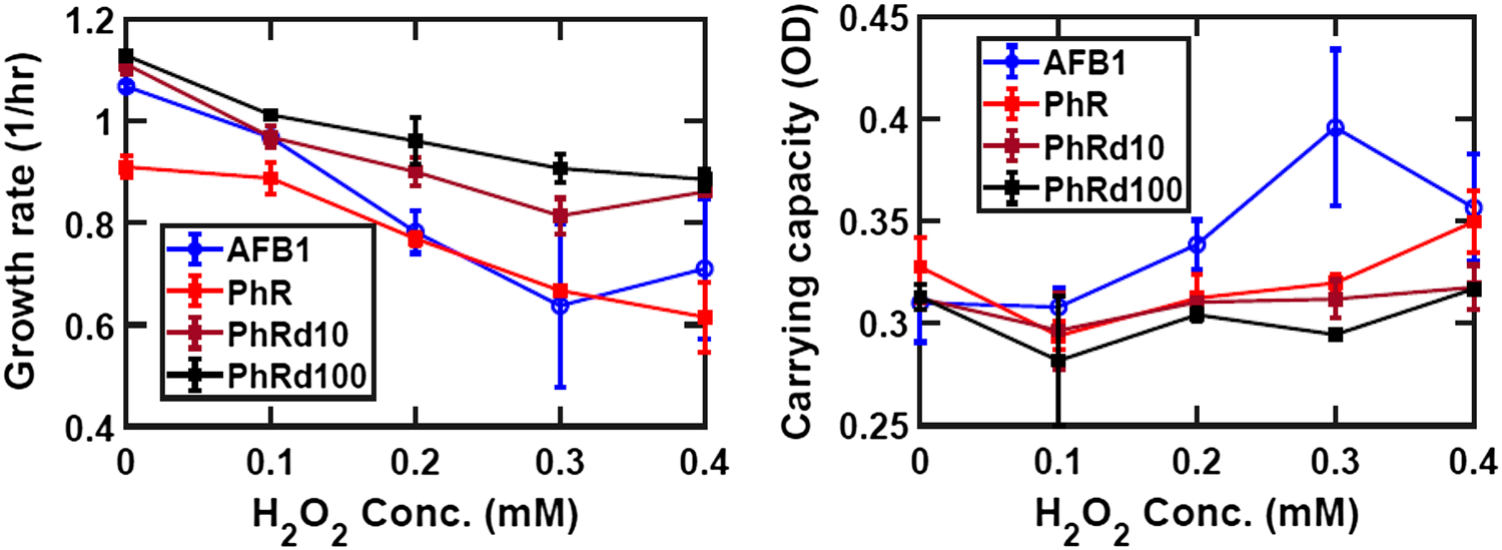
The blue line represents samples grown with 0.14 mM AFB_1_; the red line represents samples grown with 0.055 mM PhR, the maroon and black lines represent respectively tenfold and one 100-fold dilutions of the initial PhR concentration. Mean values are marked as circles, error bars are standard deviations calculated for the three experimental replicates under consideration.

The results show and increasing trend in carrying capacity with higher PhR concentrations. Results with 0.14 mM AFB_1_ exceed those of 0.055 mM PhR. Growth rate with PhR is consistent with data shown in Fig. 6 in displaying a linear drop at higher H_2_O_2_ concentrations; AFB_1_ results are within the range of those from the different PhR concentrations. We conclude that the beneficial effect of AFB_1_ on the carrying capacity of *E. coli* K12 cultures can be ascribed to the antioxidant effect of the toxin.

## Discussion

Sources of oxidants are everywhere around natural organisms: light, UV radiation, oxygen, and metals can be responsible for the formation of “environmental” RS. Eventually, oxidative stress is a condition all cells have to face to live in the presence of oxygen (and in particular singlet oxygen, ^1^O_2_)^23^. In fact, RS are, to some extent, a normal by-product of cell metabolism^24,25^. Aflatoxigenic fungi apparently have found a special way to better survive and/or prolong their survival under oxidative stress conditions.

To assess the relation between AF synthesis and oxygen content, we set up experiments under two growth conditions: a closed system (CS) and an open system (OS). In the CS, constant depletion of oxygen and accumulation of CO_2_ do not support AF synthesis. In the CS, *A. parasiticus* grows similarly to the OS condition, even if, as expected, in the CS the amount of mycelium is significantly lower than in the OS. Nevertheless, glucose consumption showed similar trends in the OS and CS conditions. From these observations, we infer that oxygen is necessary for AF synthesis, that is, oxygen is a *conditio sine qua non* for AF production, independent of mycelial growth. Hypoxia is reported^26^ to lower metabolic rates in toxigenic *Aspergilli*; this could explain the reduced fungal growth in the CS compared to the OS. Furthermore, the CS might drive *A. parasiticus* to hypoxia and therefore to a pro-glycolytic phenotype (similar to cancer’s *Warburg* effect) exploiting glucose with a reduced energetic yield. Under these conditions, RS should be present in lower amount as indicated in *A. flavus*^27^ and mammalian cells. Other studies demonstrated the key role played by endogenous^4,14,15^ and exogenous^10,28–30^ oxidative stress in AF biosynthesis. These observations point to oxidative stress and oxygen as key players for AF production in *Aspergillus* sect. Flavi. An aflatoxigenic fungus can survive in a highly oxidant environment, as demonstrated by studies in which culture media were supplemented with lipophilic epoxides^31^ or by hydroperoxides of linoleic and linolenic acids^29^. AF production in this case is highly stimulated in correlation to RS concentration^29^: the higher the RS concentration, the higher the AF production. This fungal behavior begs to inquire if there is a link between fungal survival in a toxic environment and AF production. Some authors studying the metabolism of AF producing and non-producing fungi, suggest that the formation of AF may occur as a “compensatory” response to ROS accumulation^15^. In relation to these aspects, we hypothesized an antioxidant role per se of AF and measured their antioxidant capacity. In this assay (crocin test; see Methods), the hydroperoxides generated by APAB should react with AF in lieu of crocin, if AF displayed remarkable antioxidant features^32,33^. Indeed, AFs showed, to different extents (G1 > B2 > G2 > B1), significant antioxidant capacity, comparable to other synthetic and natural antioxidants molecules^21–23^. Notwithstanding their stability under radical attack, a continuous source of ROS, such as ROO· originated by APAB, can (di)hydroxylate the furanose of AFs, as suggested by mass spectrometer results. This reaction allows the molecule to acquire two hydrophilic groups (-OH), in a hydrophilic environment. This might partly explain the antioxidant capacity of AFs. This aspect requires additional work to be verified.

Why should AFs act as antioxidants? What is the benefit for the fungus? Results on *E. coli* suggest that AF might enhance the resilience of cells to oxidative stress, prolonging their lifespan. Previous in vitro and in vivo studies indicate that *A. parasiticus* can endure high and prolonged oxidative stress conditions, during the late log/stationary phase in a culture medium^16^ as well as on stored plant seeds enriched in peroxides^32^. Recently, Linz group elegantly demonstrated that in *A. parasiticus* AFs are under the control of the oxidative stress-related factor AP-1-*like*^33^. In this model, *A. parasiticus* uses the canonical scavengers for RS (e.g. catalases) during the lag to early log phase of growth and AP-1 starts transcribing *AflR* only in the late log/stationary phase of growth, switching AF synthesis on. Within our study, it emerges that AFs are produced, and can therefore scavenge oxidants within a specific time-range: in the late log/stationary phase in which the natural antioxidant capacity of the organism (such as catalases) are probably less-active. Within this frame, evidence points at AF acting as an antioxidant to allow the fungus to extend its survival in highly oxidizing environments playing a role as another fungal adaptive strategy for life.

## Materials and methods

### Experimental design

**Figure 7:**
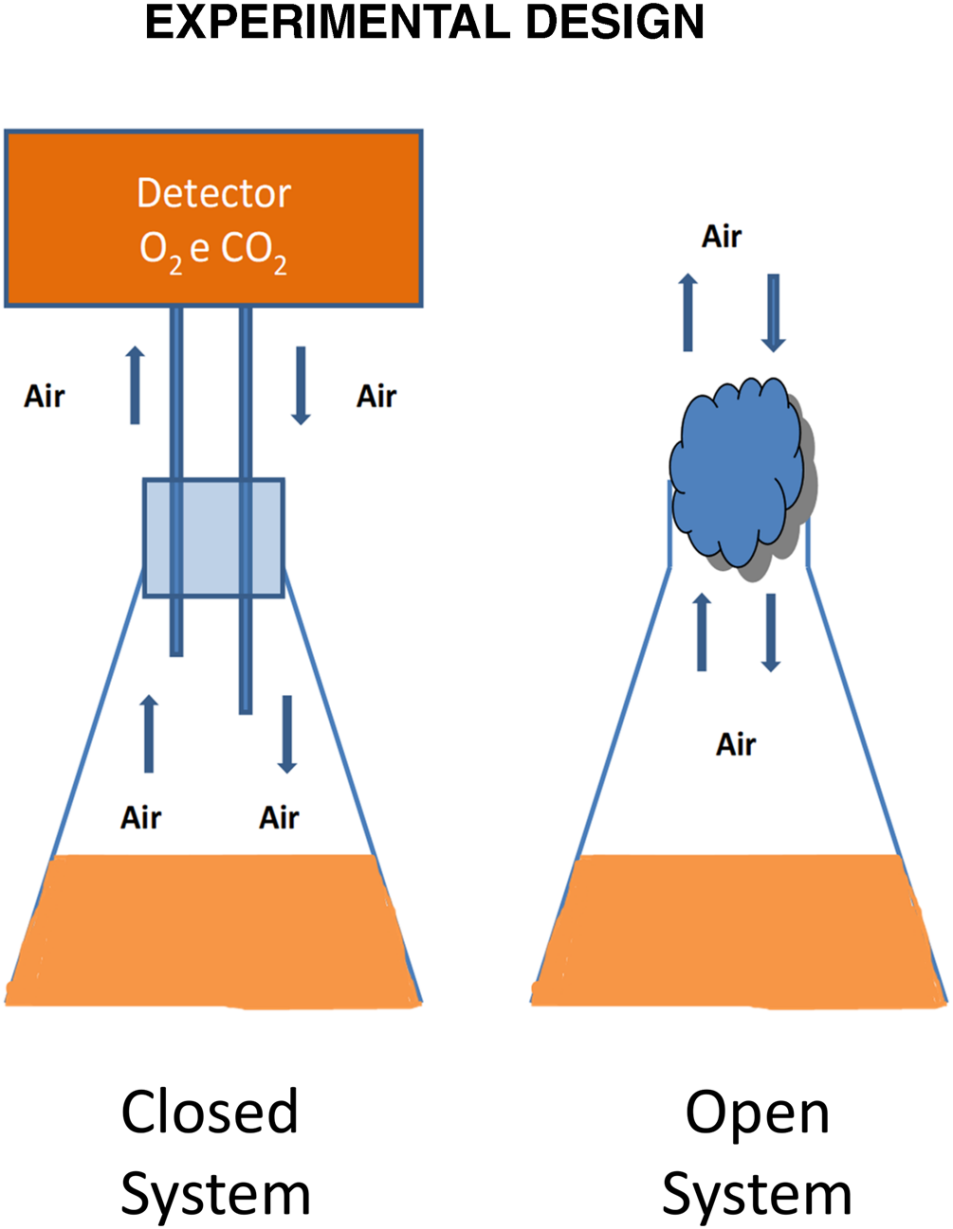
The closed system (CS) is equipped with a of O_2_ and CO_2_ detector allowing to detect during the time course of the experiment the relative quantity of the two gas. In the open system (OS) a free exchange of air between the system and the environment was established.

The experiments were carried out in two different systems of incubation (Fig. 7). An open system (OS) in which the Erlenmeyer flask was closed by cotton and a free exchange of air between the system and the environment was established. In the closed system (CS) an airflow was pushed into the Erlenmayer flask contain 20.58% of O_2_ and 0.08% of CO_2_. The flask was then hermetically closed in order to prevent air exchange between the gases produced inside and the external environment. This closed system (CS) was connected to an O_2_/CO_2_ COMBY CHECK analyzer (Dansensor Italia s.r.l., Italy) to measure the O_2_/CO_2_ ratio inside the flask during fungal growth.

### Culture conditions

*Aspergillus parasiticus* NRRL 2999, an aflatoxin producer**,** was used in these experiments. The fungal isolate was grown at 28 °C on Czapek Dox Agar (CDA) for 7 days prior to inoculation. The inoculum was performed by suspending conidia of *A. parasiticus* in 2 ml of sterile H_2_O + Triton X100 (0.1% w/v) and inoculating 1 × 10^6^ conidia in 400 mL of glucose 3% w/v and peptone 1% w/v broth (PDB). The cultures were incubated at 28 °C for 24, 48, 72, 96, and 168 h. Every 24 h, the cultures were filtered by Millipore filter (pore size 0.45 µm) and washed three times in a saline solution (NaCl 0.9% w/v). The mycelium was dried at 80 °C for 48 h and then weighed. The consumption of glucose has been estimated with Boehringer Mannheim (Germany) D-glucose kit.

### Determination of hydrophilic and lipophilic antioxidant capacity

The antioxidant capacity of each single AFs has been estimated by crocin bleaching inhibition method^36^ This method is based on the bleaching of crocin as a result of its oxidation by a source of radicals, APAB ([2,2'-Azobis(2-methylpropionamidine) dihydrochloride] and AMVN (2,2’-azobis (2,4-dimethylvaleronitrile). The reaction is monitored by recording, for ten minutes, the corresponding decrease of absorbance at 443 nm. The reaction with crocin alone gives us the bleaching rate V_0;_ when an antioxidant or pseudo-antioxidant is added, it reacts with the free radicals and, as a consequence, crocin bleaching rate (V_a_) is reduced, according to the competitive reaction equation:

$$\frac{{V_{0} }}{{V_{a} }} = 1 + \frac{{K_{a} }}{{K_{c} }}\frac{[Pseudoantioxidant]}{{[Crocin]}}$$

where *K*_*c*_ and *K*_*a*_ are the respective absolute second order constants. The slope *K*_*a*_*/K*_*c*_ has been calculated by means of the Pseudo-antioxidant/crocin*vs* V_0_/V_a_ linear regression plot. Its value indicates the relative capacity (antioxidant capacity) of different molecules to interact with the ROO radicals. APAB 40 mM (Waco Chem, Richmond VA, USA) and crocin 0.24 mM were added to H_2_O, and bleaching rate of crocin was determined after 10 min from the start of the reaction. The reaction was carried out at 40 °C. Blank samples were run to rule out spectral interferences between compounds and crocin. All hydrophilic extracts corresponding to each sample under investigation were tested. Each kinetic analysis was compared with kinetic crocin bleach containing only APAB (with bleaching rate V_0_) and used for the calculations according to the competitive reaction equation. The same method was used for the measurement of the lipophilic antioxidant capacity, using 2,2’-azobis (2,4-dimethylvaleronitrile) (AMVN) (Waco Chem, Richmond VA, USA) as a free radical source^34,35^. Solvents were from now Merck KGaA (St Louis, MO, USA).

### Aflatoxin analysis

Aflatoxins were extracted following the method described in Zaccaria et al.^10^ from 2 mL of culture filtrates of *A. parasiticus* grown in OS as well as in CS conditions. For the LC MS/MS aflatoxins analysis, a calibrated solution of the unlabeled AFB_1_, B_2_, G_1_ and G_2_ was prepared to cover a concentration range of 0.005 to 50 ng/mL. Chromatographic separation and MRM quantification of the four aflatoxins was carried out with an Agilent 1200 Infinity HPLC system coupled to an Agilent G6420 Triple Quadrupole mass spectrometer as reported in^36^.

### Aflatoxin oxidation products characterization

For the discovery of AF degradation products, four solutions containing different aflatoxins (AF B_1_, B_2_, G_1_ or G_2_; 0.2 mg each) and APAB (4 mg) have been dissolved in 4 mL of water and kept at 40 °C for 30 min. Then, AFs have been extracted from 1 mL aliquots of the solution (as reported below) for analysis. LC/MS analysis was performed with the following equipment and reagents: Kinetex column 2.6 µM EVO C18, 100 × 2.1 mm; mobile phase A: Water 5 mM Ammonium Acetate, 0.5% Acetic Acid; mobile phase B: Methanol, 5 mM Ammonium Acetate, 0.5% Acetic Acid, at a flow rate of 350 µl/min, and with UV wavelengths at 354 and 360 nm (Table 1).

**Table 1:** Gradient scheme.

Time (min)	%A	%B
Initial	90	10
3	90	10
10	30	70
10.1	10	90
12	10	90
12.1	90	10
15	90	10

The eluent from the column was directed into the electrospray source of an Agilent 6220 TOF mass spectrometer operated in positive ionization mode. Data was converted into mzML file format and analyzed using MZMine software.

### In vitro test of aflatoxins antioxidant capacity

*Escherichia coli* K12 cell cultures were exposed to different concentrations of hydrogen peroxide (H_2_O_2,_ Thermo-Fisher Scientific, Waltham, MA, USA) in the presence of AFB_1_. *E. coli* was selected for the purpose of this experiment because it lacks the enzyme Cytochrome P450, whose interaction with AFB_1_ is the mechanism through which the toxin causes its deleterious effect to cells^37,38^. Therefore, in this experimental context, the antioxidant effect of AF does not come with a viability cost for the bacterial cells. Defined medium “Z” (KH_2_PO_4_ 1.5 g/L; K_2_HPO_4_ × 3H_2_O 3.8 g/L; (NH_4_)_2_SO_4_ 1.3 g/L; Na citrate × 2H_2_O 3.0 g/L; Glucose 4.0 g/L; 1 M MgCl_2_; 1 M CaCl_2_, pH 7.2) was used to grow *E.coli* K12 strain MG1655 at 37 °C in agitation. AFB_1_ (Cayman Chemical Company, Ann Arbor, MI, USA) was dissolved in methanol and added to the culture medium at the concentration of 20 µg/mL. H_2_O_2_ was added to the culture medium at thirteen different concentrations from 0.1 to 0.7 mM (0.05 increments). Control samples were prepared by adding the respective amount of methanol without the toxin of the AFB_1_-supplemented cultures, and loaded on the same multiwell plate. Each combination of treated and control samples counted ten replicates for all the different concentrations of peroxide tested. For the purpose of data analysis, the most peripheral wells were excluded to rule out evaporation-related variability. The cell viability test was run on a 384 multiwell plate on a Synergy™ Mx Multi-Mode Microplate Reader (Agilent Technologies, Santa Clara, CA, USA). Cellular growth was assayed using absorbance readings at 600 nm (OD_600_). AFB_1_ concentration was monitored via fluorescence assay (ex. 380 nm, em. 480 nm). Experimental time was set at 24 h; absorbance and fluorescence measurements were obtained every 4 min for a total of 361 reads. Growth rate was defined as the rate of exponential increase in OD values in the range between 0.05 and 0.2 and calculated in Matlab using polyfit. A second experiment with *E. coli* K12 cultures was performed including alternatively AFB_1_ (Cayman Chemical Company, Ann Arbor, MI, USA) and PhR (Thermo-Fisher Scientific, Waltham, MA, USA) as antioxidants. The cell viability test was run on a 96 multiwell plate on a Synergy™ H4 Multi-Mode Microplate Reader (Agilent Technologies, Santa Clara, CA, USA). Cellular growth was assayed using absorbance readings at 600 nm (OD_600_). Experimental time was set at 24 h; absorbance measurements were obtained every 5 min for a total of 289 reads. H_2_O_2_ was added to the culture medium at five different concentrations from 0 to 0.4 mM (0.1 increments). Growth rate was defined as the rate of exponential increase in OD values in the range between 0.05 and 0.2 and calculated in Matlab using (fit_logistic.m); edge wells, potentially affected by evaporation, were discarded from the analysis.

## References

[1] Kumar, P., Mahato, D. K., Kamle, M., Mohanta, T. K., Kang, S. G. Aflatoxins. A global concern for food safety, human health and their management*. Front. Microbiol*. **7**, 2170 (2017). 10.3389/fmicb.2016.02170 PMC5240007 28144235

[2] Battilani, P. *et al*. Aflatoxin B1 contamination in maize in Europe increases due to climate change. *Sci. Rep.* **6**, 95325: 24328 (2016). 10.1038/srep24328 PMC4828719 27066906

[3] Fox EM, Howlett BJ (2008). Secondary metabolism: Regulation and role in fungal biology. Curr. Opin. Microbiol..

[4] Reverberi M, Ricelli A, Zjalic S, Fabbri AA, Fanelli C (2010). Natural functions of mycotoxins and control of their biosynthesis in fungi. Appl. Microbiol. Biotechnol..

[5] Nicoletti R, De Stefano S, Trincone A, Marziano F (2004). Antagonism against *Rhizoctoniasolani* and fungi toxic metabolite production by some *Penicillium* isolates. Mycopathologia.

[6] Boddu J, Cho S, Muehlbauer GJ (2007). Transcriptome analysis of trichothecene-induced. Mol. Plant Micr. Interact..

[7] Desmond OJ (2008). The Fusarium mycotoxin deoxynivalenol. Mol. Plant Pathol..

[8] Sugui JA (2007). Gliotoxin is a virulence factor of *Aspergillus fumigatus*: *gliP* deletion attenuates virulence in mice immunosuppressed with hydrocortisone. Eukaryot. Cell.

[9] Paciolla C, Dipierro N, Mulè G, Logrieco A, Dipierro S (2004). The mycotoxins beauvericin and T2 induce cell death and the alteration to the ascorbate metabolism in tomato protoplast. Physiol. Mol. Plant Pathol..

[10] Zaccaria M (2015). Menadione-induced oxidative stress re-shapes the oxylipin profile of *Aspergillus flavus* and its lifestyle. Toxins.

[11] Roze LV (2015). Aflatoxin biosynthesis is a novel source of reactive oxygen species A potential redox signal to initiate resistance to oxidative stress?. Toxins.

[12] Okoth, S. *et al*. Genetic and toxigenic variability within *Aspergillus flavus* population isolated from maize in two diverse environments in Kenya. *Front. Microbiol.* **9**, 57 (2018). 10.3389/fmicb.2018.00057 PMC5790802 29434580

[13] Bennett JW, Bennett JW, Ciegler A (1983). Differentiation and secondary metabolites in Mycelia fungi. Secondary Metabolism and Differentiation in Fungi.

[14] Jayashree T, Subramanyam C (2000). Oxidative stress is a prerequisite for aflatoxin production by *Aspergillus parasiticus*. Free Rad. Biol. Med..

[15] Narasaiah KV, Sashidar RB, Subramanyam C (2006). Biochemical analysis of oxidative stress in the production of aflatoxin and its precursor intermediates. Mycopathologia.

[16] Reverberi M, Zjalic S, Ricelli A, Fabbri AA, Fanelli C (2006). Oxidant-antioxidant balance in Aspergillus parasiticus affects aflatoxin biosynthesis. Mycotoxin Res..

[17] Gow-Chin Y, Yung-Chi C (1999). Medium optimization for the production of antioxidants from *Aspergillus candidus*. J. Food Prot..

[18] Di Majo D (2005). Flavonones in citrus fruit: Structure-antioxidant activity relationships. Food Res. Int..

[19] Finotti E, Di Majo D (2003). Influence of solvents on the antioxidant property of flavonoids. Nahrung/Food.

[20] Finotti E, D’Ambrosio M, Paoletti F, Vivanti V, Quaglia G (2000). Synergistic effect of -tocopherol, -sitosterol and squalene on antioxidant activity assayed by crocin bleaching method. Nahrung.

[21] Halliwell D, Gutteridge JMC, Halliwell D, Gutteridge JMC (2007). Oxygen is a toxic gas-an introduction to oxygen toxicity and reactive species. Free Radicals in Biology and Medicine.

[22] Guengerich FP, Arneson KO, Williams KM, Deng Z, Harris TM (2002). Reaction of aflatoxin B_1_ oxidation products with lysine. Chem. Res. Toxicol..

[23] Mao J (2016). Structure identification and toxicity assessment of the degradation products of aflatoxin B_1_ in peanut oil under UV irradiation. Toxins.

[24] Dowling DK, Simmons LW (2009). Reactive oxygen species as universal constraints in the life-history evolution. Proc. Res. Soc..

[25] Halliwell D, Gutteridge JMC, Halliwell D, Gutteridge JMC (2007). Cellular responses to oxidative stress: Adaptation, damage, repair, senescence and death. Free Radicals in Biology and Medicine.

[26] Shimizu M (2018). NAD/NADH homeostasis affects metabolic adaptation to hypoxia and secondary metabolite production in filamentous fungi. Biosci. Biotechnol. Biochem..

[27] Damiani E (2018). Modulation of oxidative status by normoxia and hypoxia on cultures of human dermal fibroblasts: How does it affect cell aging?. Oxid. Med. Cell. Longev..

[28] Harman D (1956). Aging a theory based on free radical and radiation chemistry. J. Gerontol..

[29] Fanelli C, Fabbri AA, Finotti E, Fasella P, Passi S (1984). Free radicals and aflatoxin biosynthesis. Experientia.

[30] Fabbri AA, Fanelli C, Panfili G, Passi S, Fasella P (1983). Lipoperoxidation and aflatoxin biosynthesis by *Aspergillus parasiticus* and *A. flavus*. J. Gen. Microbiol..

[31] Reverberi M (2008). Modulation of antioxidant defense in *Aspergillus parasiticus* is involved in aflatoxin biosynthesis: A role for the Ap*yapA* gene. Eukaryot. Cell..

[32] Fanelli C, Fabbri AA, Finotti E, Passi S (1983). Stimulation of aflatoxin biosynthesis by lipophilic epoxides. J. Gen. Microbiol..

[33] Hong S-Y, Roze LV, Linz JE (2013). Oxidative stress-related transcription factors in the regulation of secondary metabolism. Toxins..

[34] Bors W, Michael C, Saran M (1984). Inhibition of bleaching of the carotenoid crocin. A rapid test for quantifying antioxidant activity. Biochem. Biophys. Acta.

[35] Ordoudi SA, Tsimidou MZ (2006). Crocin bleaching assay step by step: Observations and suggestions for an alternative validated protocol. J. Agric. Food Chem..

[36] Sobolev AP (2018). A multi-methodological approach in the study of Italian PDO “Cornetto di Pontecorvo” red sweet pepper. Food Chem..

[37] Eaton DL, Groopman JD (1994). The Toxicology of Aflatoxins: Human Health, Veterinary, and Agricultural Significance.

[38] Guengerich FP, Martin MV, Guo Z, Chun YJ (1996). Purification of functional recombinant P450s from bacteria. Methods Enzimol..

